# Effect of Sulfate on Carbon Monoxide Conversion by a Thermophilic Syngas-Fermenting Culture Dominated by a *Desulfofundulus* Species

**DOI:** 10.3389/fmicb.2020.588468

**Published:** 2020-11-16

**Authors:** Joana I. Alves, Michael Visser, Ana L. Arantes, Bart Nijsse, Caroline M. Plugge, M. Madalena Alves, Alfons J. M. Stams, Diana Z. Sousa

**Affiliations:** ^1^Centre of Biological Engineering, University of Minho, Braga, Portugal; ^2^Laboratory of Microbiology, Wageningen University & Research, Wageningen, Netherlands; ^3^Laboratory of Systems and Synthetic Biology, Wageningen University & Research, Wageningen, Netherlands

**Keywords:** syngas fermentation, carbon monoxide, carboxydotroph, sulfate-reducing bacterium, metagenomics

## Abstract

A syngas-degrading enrichment culture, culture T-Syn, was dominated by a bacterium closely related to *Desulfofundulus australicus* strain AB33^T^ (98% 16S rRNA gene sequence identity). Culture T-Syn could convert high CO concentrations (from pCO ≈ 34 kPa to pCO ≈ 170 kPa), both in the absence and in the presence of sulfate as external electron acceptor. The products formed from CO conversion were H_2_ and acetate. With sulfate, a lower H_2_/acetate ratio was observed in the product profile, but CO conversion rates were similar to those in the absence of sulfate. The ability of *D. australicus* strain AB33^T^ to use CO was also investigated. *D. australicus* strain AB33^T^ uses up to 40% CO (pCO ≈ 68 kPa) with sulfate and up to 20% CO (pCO ≈ 34 kPa) without sulfate. Comparison of the metagenome-assembled genome (MAG) of the *Desulfofundulus* sp. from T-Syn culture with the genome of *D. australicus* strain AB33^T^ revealed high similarity, with an ANI value of 99% and only 32 unique genes in the genome of the *Desulfofundulus* sp. T-Syn. So far, only *Desulfotomaculum nigrificans* strain CO-1-SRB had been described to grow with CO with and without sulfate. This work further shows the carboxydotrophic potential of *Desulfofundulus* genus for CO conversion, both in sulfate-rich and low-sulfate environments.

## Introduction

Syngas, which is characteristically a mixture composed of CO, H_2_, and CO_2_, can be used as a source of electrons in a variety of biotechnological applications, such as the production of chemicals (e.g., acetate and ethanol) (Köpke et al., 2010; Daniell et al., 2012; Molitor et al., 2016; Grimalt-Alemany et al., 2018) or the recovery of valuable resources (e.g., metal recovery from sulfate-rich wastewaters) (Parshina et al., 2010). Several mesophilic and thermophilic microorganisms, belonging to different physiological clusters (i.e., acetogens, hydrogenogens, methanogens, or sulfate reducers), have been shown to use CO (Henstra et al., 2007; Sokolova et al., 2009; Diender et al., 2015). These microorganisms are termed as carboxydotrophs and are characterized for possessing carbon monoxide dehydrogenase (CODH) activity (Henstra et al., 2007).

Acetogens and sulfate-reducing bacteria (SRB) use the Wood–Ljungdahl pathway to convert CO to acetyl-CoA, further yielding acetate and allowing energy conservation in the form of ATP (Diender et al., 2015). In the presence of sulfate, most SRB convert CO to CO_2_ and H_2_ (water–gas shift reaction, catalyzed by CODH) and, subsequently, use H_2_ for sulfate reduction and energy conservation (Rabus et al., 2006).

In former studies by Alves et al. (2013a), a syngas-utilizing culture (T-Syn culture) was obtained, which was dominated by a bacterial species closely related to *Desulfofundulus australicus* (formerly named *Desulfotomaculum australicus*, emend. Watanabe et al., 2018). This T-Syn culture was enriched with CO, in the absence of sulfate, and converted syngas (total pressure ≈ 170 kPa; 50% CO in the gas mixture composed of CO:H_2_:CO_2_) to mainly acetate. Bacteria of the genus *Desulfofundulus* are known to perform sulfate reduction (Watanabe et al., 2018), but previously, another SRB, *Desulfotomaculum nigrificans* strain CO-1-SRB, had been described as capable of growing on 100% CO (total pressure at 120–180 kPa) both in the absence or in the presence of sulfate (Davidova et al., 1994; Parshina et al., 2005b; Visser et al., 2014). In addition, several species of *Desulfofundulus* and *Desulfotomaculum* genera are reported to convert CO to acetate and hydrogen with sulfate as electron acceptor (Davidova et al., 1994; Plugge et al., 2002; Henstra et al., 2007; Parshina et al., 2010). Carbon monoxide utilization by thermophilic SRB has been studied (Oelgeschlager and Rother, 2008; Sokolova et al., 2009; Parshina et al., 2010) and it was shown that thermophilic sulfate reducers tolerate CO better than mesophilic SRB (Parshina et al., 2005a). Besides *D. nigrificans* strain CO-1-SRB being known by its ability to grow with 100% of CO (total pressure at 120–180 kPa), some other thermophilic SRB, e.g., *Desulfofundulus thermobenzoicus* subsp. *thermosyntrophicus* or *Desulfofundulus kuznetsovii* (formerly members of *Desulfotomaculum* genus, emend. Watanabe et al., 2018), can grow well at medium to high CO concentrations (e.g., 20–70% in the headspace; total pressure at 100 kPa) (Davidova et al., 1994; Parshina et al., 2005a,b; Henstra et al., 2007; Visser et al., 2014).

In this work, the syngas-degrading T-Syn culture was used to investigate the effect of sulfate on the conversion of different CO concentrations. A physiological and genomic comparison between the *Desulfofundulus* sp. from T-Syn culture and the type strain *D. australicus* strain AB33^T^ was also performed.

## Materials and Methods

### Source of Thermophilic Cultures

Enrichment of T-Syn culture was described elsewhere (Alves et al., 2013a). Culture T-Syn was kept active by successive transfers in anaerobic basal medium (see composition in section “Medium Composition and Cultivation”) and a gas phase of 50% CO [serum bottles of 120 mL containing 50 mL of medium and headspace filled with 170 kPa CO + N_2_ (50/50%)]. *D. australicus* strain AB33^T^ was obtained from the German Collection of Microorganisms and Cell Cultures (DSM 11792; DSMZ, Braunschweig, Germany).

### Medium Composition and Cultivation

A phosphate-buffered (20 mM) mineral salt medium (pH 7.0) was used, containing the following components (per liter): Na_2_HPO_4_, 1.63 g; NaH_2_PO_4_, 1.02 g; resazurin, 0.5 mg; NH_4_Cl, 0.3 g; CaCl_2_.2H_2_0, 0.11 g; MgCl_2_.6H_2_0, 0.10 g; NaCl, 0.3 g. 1 mL from the acidic and alkaline stock solutions of trace elements and 0.2 ml of vitamin stock were also added (per liter). These solutions were prepared as described previously by Stams et al. (1993). Before inoculation, the medium was reduced with ≈ 0.8 mM Na_2_S.7–9H_2_O (final concentration). T-Syn culture was incubated with five different CO concentrations in the headspace: 20%, 40%, 60%, 80%, and 100% CO (pCO/P; *P* = 170 kPa). Each condition of CO concentration was tested with and without sulfate as external electron acceptor, resulting in ten different conditions; N_2_ was used to pressurize the bottles’ headspace for CO percentages lower than 100% (to achieve identical initial pressure in all the assays, 170 kPa). The freeze-dried *D. australicus* strain AB33^T^ was firstly activated with lactate (20 mM) as carbon and energy source and sulfate as electron acceptor (20 mM) and incubated at 55°C; after activation, this culture was incubated with carbon monoxide and sulfate, as an adaptation step. Subsequently, the *D. australicus* strain AB33^T^ was incubated following the same testing conditions as for T-Syn culture: 5 different CO concentrations in the headspace (20%, 40%, 60%, 80%, and 100% CO; with and without sulfate as external electron acceptor; total pressure 170 kPa). When sulfate was used as final electron acceptor, sodium sulfate was added to the medium from sterile anoxic stock solutions (1 M) to a final concentration of 20 mM. All the growth experiments were done in triplicate. All the bottles were incubated while stirring (100 rpm) in the dark, at 55°C. All the inoculations, transfers, and the addition of stock solutions were performed aseptically using sterile syringes and needles.

### Analytical Methods

Volatile fatty acids were quantified using a Jasco HPLC (Tokyo, Japan) equipped with a Chrompack column (6.5 mm× 30 mm) and a UV detector (210 nm). A flow rate of 0.6 mL min^–1^ was used with sulfuric acid (0.01 N) as mobile phase and column temperature set at 60°C. Gaseous compounds (CO, CO_2_, H_2_) were analyzed by gas chromatography on a Bruker Scion 456-GC (Billerica, MA, United States) with a thermal conductivity detector. CO_2_ was analyzed with a BR-QPLOT column (30 m length, 0.53 mm internal diameter; film thickness, 20 μm), in which helium was used as carrier gas at a flow rate of 15 mL min^–1^, and the temperatures in the injector, column, and detector were 60, 35, and 130°C, respectively. CO and H_2_ were analyzed with a Molsieve packed column (13 × 80/100, 2 m length, 2.1 mm internal diameter). Argon was used as carrier gas at a flow rate of 20 mL min^–1^, and temperatures in the injector, column, and detector were 100, 35, and 130°C, respectively. Total dissolved sulfide was determined using a standard kit (Hach Lange, Düsseldorf, Germany). Cultures were monitored by microscopic examination during growth with CO (Olympus CX41, Tokyo, Japan).

### DNA Isolation and 16S rRNA Gene Cloning and Sequencing

DNA from T-Syn culture incubated with 100% CO (total pressure 170 kPa), with and without sulfate, was extracted using a FastDNA SPIN kit for soil (MP Biomedicals, Solon, OH, United States) in accordance with the manufacturer’s instructions. Bacterial 16S rRNA genes were amplified by PCR using a Taq DNA polymerase kit (Thermo Fisher Scientific, Waltham, MA, United States); reaction mixtures and PCR programs used were as described elsewhere (Alves et al., 2013a). Primer set Bact27f/Uni1492r was used for 16S rRNA gene amplification for cloning and sequencing purposes (Lane, 1991; Nübel et al., 1996). Cloning of PCR amplicons, purification, transformation, and selection of transformants for DNA sequence analysis were performed as previously described in detail by Alves et al. (2013a). Sequencing reactions were performed at Macrogen (Amsterdam, Netherlands) using pGEM-T vector-targeted sequencing primers SP6 and T7 and internal tailored primers, if needed. Partial sequences were assembled by using the Contig Assembly Program (CAP) application included in the BioEdit v7.0.9 software package (Huang, 1992; Hall, 1999). Consensus sequences obtained were checked for potential chimera artifacts using Bellerophon software (Huber et al., 2004). Similarity searches for the 16S rRNA gene sequences derived from the clones were performed using the National Center for Biotechnology Information (NCBI, Bethesda, MD, United States) BLAST search program within the GenBank database^1^ (Altschul et al., 1990). The 16S rRNA gene sequence predominant among cloned sequences in the library from T-Syn enrichment culture was compared with sequences from the database and represented in a phylogenetic tree. This tree was constructed with the MEGA X software package (Kumar et al., 2018), using the Neighbor-joining method (Saitou and Nei, 1987); the evolutionary distances were computed using the p-distance method (Nei and Kumar, 2000).

### DNA Isolation for Genome Sequencing, Metagenome Binning, and Annotation

Genomic DNA from T-Syn culture growing on CO (with sulfate) was isolated using a Gram-positive genomic DNA isolation kit (Epicentre, Madison, WI, United States). Sequencing of the metagenome of T-Syn was performed by using Illumina MiSeq sequencing technology. The paired-end sequence data was checked for quality by using FastQC (Andrews, 2014). The A5-miseq v20141120 pipeline was used to trim and correct the reads but not for the assembly (Coil et al., 2015). Before assembly, the reads were extended using FLASH v1.2.11 (Magoc and Salzberg, 2011), with settings of a minimum overlap of 10 and maximum of 250 bp. This resulted in an optimal Kmer length of 155 for assembly for T-Syn with merged unpaired and unmerged paired-end reads, which were subsequently used by the *de novo* assembler Ray v 2.3.1. The assembly was improved by using Bowtie2 v 2.2.4 (Langmead and Salzberg, 2012), to correct the merged and unmerged reads, and Pilon v 1.10 (Walker et al., 2014). Samtools v 1.1 was used to convert SAM files to BAM format and for indexing (Li et al., 2009). The final assembly resulted 53 for T-Syn, with an N50 length of 95655 bp. The scaffolds were submitted to the SAPP framework for annotation (Koehorst et al., 2016). The genome of *D. australicus* strain AB33^T^ is publicly available at the Integrated Microbial Genomes and Microbiomes server (Joint Genome Institute, Walnut Creek, CA, United States). The scaffolds of *D. australicus* strain AB33^T^ were downloaded and were submitted to the same gene prediction method. For T-Syn culture, the metagenomic binning Metawrap v1.2 (Docker version) was used with the binning tools MaxBin2, MetaBat2, and Concoct (Alneberg et al., 2014; Wu et al., 2016; Uritskiy et al., 2018; Kang et al., 2019). Bins were checked for completion and contamination according to the methodology described by Bowers et al. (2017), and further taxonomic affiliation was done based on Bowers et al. (2017).

### Genome Comparison

The draft metagenome-assembled genome (MAG) of the *Desulfofundulus* sp. in culture T-Syn and the genome of *D. australicus* strain AB33^T^ were compared by using National Center for Biotechnology Information (NCBI, Bethesda, MD, United States) BLAST. The nucleotide sequences of the T-Syn and *D. australicus* strain AB33^T^ genes were converted to amino acid sequences. Subsequently, these sequences were used for a bidirectional BlastP analysis, using standard settings, and hits were filtered for an *e*-value of <0.0001 and limited to showing one hit. Genes that were present in the MAG of *Desulfofundulus* sp. T-Syn and not in *D. australicus* strain AB33^T^ and vice versa were compared to the non-redundant protein sequences database to determine homologs. Additionally, several proteins of interest were investigated in more detail by using them as a query for a separate BlastP analysis. The average nucleotide identity (ANI) of shared genes was calculated using species v1.2.1 to determine the phylogenetic relation of *Desulfofundulus* sp. T-Syn with *D. australicus* strain AB33^T^. A ANI value of 95% corresponds to a 70% DNA-DNA hybridization level, defined as the threshold for the definition of a species (Richter and Rosselló-Móra, 2009).

## Results and Discussion

### CO Conversion in the Presence and Absence of Sulfate by T-Syn Culture

T-Syn culture was enriched from thermophilic (55°C) anaerobic sludge with different syngas (CO:H_2_:CO_2_) mixtures, increasing CO partial pressure, from 5 to 50%, 170 kPa, without sulfate (Alves et al., 2013a). Subsequently, CO partial pressure within the syngas mixture was gradually increased [from 50 to 100% (pure CO); 170 kPa] and T-Syn culture grown with syngas was mainly composed of a bacterium related to *Desulfofundulus* (≈ 70% of the bacterial clones sequenced) (Alves et al., 2013a). Members of *Desulfofundulus*, *Desulfotomaculum*, and *Moorella* genera are known to use syngas/CO. They can produce acetate, as observed for *D. kuznetsovii* and *Moorella thermoacetica*, or produce hydrogen, such as *D. nigrificans* and *Moorella stamsii* (Parshina et al., 2005b; Sipma et al., 2006; Jiang et al., 2009; Alves et al., 2013b; Visser et al., 2014; Watanabe et al., 2018). T-Syn culture was further used in this study to test the effect of the presence or absence of sulfate on CO utilization. T-Syn culture was transferred to medium with CO concentrations up to 100% in the headspace (total pressure 170 kPa). The microbial community of T-Syn culture growing with 100% CO, with and without sulfate, was characterized by cloning and sequencing of the 16S rRNA gene to evaluate if there were changes in relation to the inoculum T-Syn [grown on 50% CO and previously described by Alves et al. (2013a)]. 95% of the total 167 clones sequenced (75 clones retrieved from the T-Syn incubations without sulfate, and 92 clones from T-Syn with sulfate) were affiliated with bacteria from the genus *Desulfofundulus*, more specifically with *D. australicus* strain AB33^T^ (16S rRNA gene sequences identity of ∼98%) (Table 1). Dominance of one single morphotype in culture T-Syn was also confirmed by microscopic observation.

**TABLE 1:** Phylogenetic affiliation of cloned 16S rRNA gene sequences corresponding to the predominant clones retrieved from clone library of T-Syn culture (100% CO; *P*_total_ = 170 kPa) under different growth conditions: clone WS, with sulfate; clone WOS, without sulfate.

Clone ID	Phylum^a^/Class^a^	Closest relatives^b^	16S rRNA gene identity^b^ (%)	Accession number
cl1_WS (1585 bp)	*Firmicutes*/*Clostridia*	*Desulfotomaculum* sp., clone SYN_1 (HF562211)	99.4%	HG380098
		*Desulfofundulus australicus* strain AB33^T^ (NR037008)	98.4%	
cl1_WOS (1596 bp)	*Firmicutes*/*Clostridia*	*Desulfotomaculum* sp., clone SYN_1 (HF562211)	98.5%	HG380099
		*Desulfofundulus australicus* strain AB33^T^ (NR037008)	97.4%	

*^*a*^ Classified using the RDP Naïve Bayesian Classifier.^*b*^ Results of sequence alignment by using BLAST toward the NCBI nucleotide database of partial 16S rRNA gene sequences.*

**FIGURE 1:**
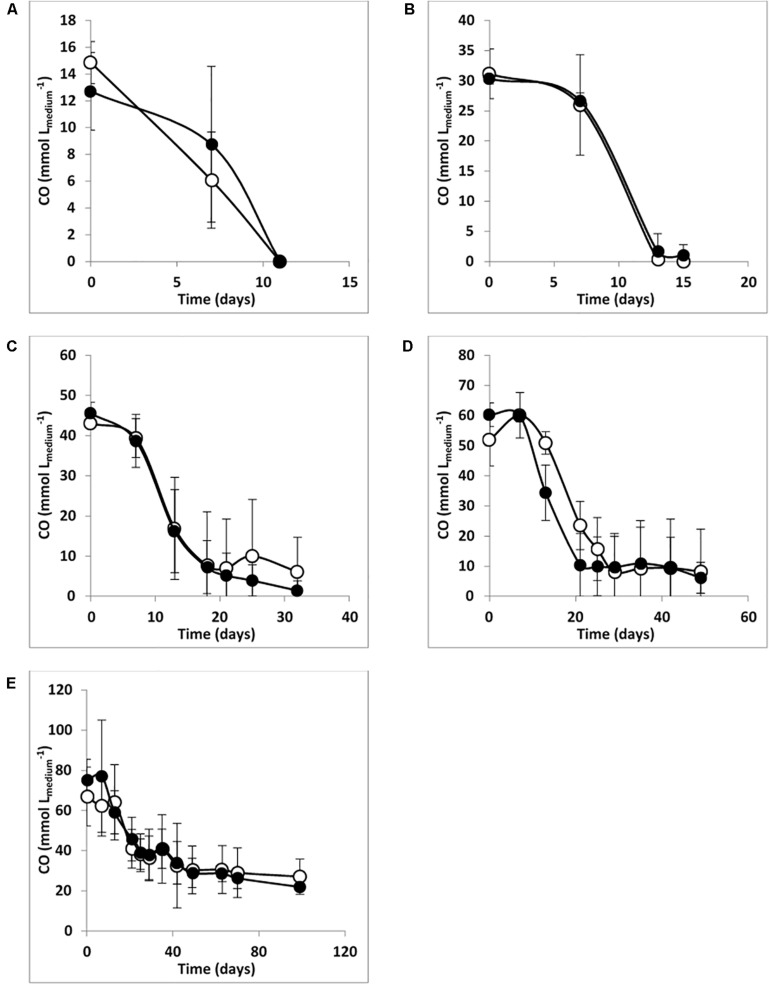
CO degradation by enrichment culture T-Syn with **(A)** 20%, **(B)** 40%, **(C)** 60%, **(D)** 80%, and **(E)** 100% of CO, *P*_total_ = 170 kPa. Open symbols: medium without sulfate; filled symbols: medium with sulfate (≈ 20 mM). The bars indicate maximum and minimum values measured (*n* = 3).

The absence/presence of sulfate does not seem to affect the predominance of the *Desulfofundulus* sp. in culture T-Syn (which was ∼95% of clone sequences in libraries from WS and WOS enrichments), pointing toward the hypothesis that this organism is the main player in CO conversion by T-Syn. Figure 1 also shows that the presence or the absence of sulfate did not significantly affect the CO conversion rates for each CO concentration studied.

These results resemble the findings of Parshina et al. (2005b) that showed for the first time an SRB, *D. nigrificans* strain CO-1-SRB [formerly named *Desulfotomaculum carboxydivorans*, emend. Visser et al. (2014)], that can grow and use CO as an electron donor at concentrations up to 100% (180 kPa), in the presence and in the absence of sulfate. During growth and CO conversion by T-Syn culture, H_2_ and acetate were the main products identified, both in the presence and in the absence of sulfate (Table 2). Specifically, in the absence of sulfate, a higher H_2_/acetate ratio was observed. Higher CO partial pressures (up to 80% in the headspace; 170 kPa) caused a direct increase in H_2_ production, for example 10.6 mmol L_medium_^–1^, when growing with 20% CO (170 kPa), and 37.9 mmol L_medium_^–1^ for 80% CO (170 kPa). The exception was for the experiments with 100% CO in the headspace (170 kPa), where almost no hydrogen was formed (Table 2). The same observation was obtained in the parallel experiments (also with 100% CO), when sulfate was added to the medium (Table 2). These results suggest that 100% CO in the gas phase (170 kPa) inhibits hydrogenase activity. This reaction, in which CO binds at the active site of hydrogenases inhibiting the catalysis of these enzymes, is well studied (Purec et al., 1962; Bennett et al., 2000; Matsumoto et al., 2011). Several examples of this inhibitory effect of hydrogenase activity in anaerobic microorganisms, such as *Eubacterium limosum*, *Desulfovibrio vulgaris*, and *Desulfofundulus thermobenzoicus* subsp. *thermosyntrophicus* have been described (Daniels et al., 1977; Genthner and Bryant, 1982; Berlier et al., 1987; Parshina et al., 2005a; Watanabe et al., 2018). As already stated, acetate and hydrogen production occurs in both conditions studied, which is represented by equations 1 and 3, respectively. This acetate and/or hydrogen formed can then be used for sulfate reduction (equation 2 and equation 4, respectively). A direct CO oxidation coupled to sulfate reduction (equation 5) is also possible [5]. Additionally, T-Syn culture produces mainly acetate in the presence of sulfate, indicating that acetate is not the electron donor for sulfate reduction.

(1) 4CO+2HO2→CHCOO3+-2CO+2H+

(2) CHCOO3+-2H++SO→42-2CO+22HO2+HS-

(3) CO+HO2→H+2CO2

(4) 4H+2SO+42-H→+HS+-4HO2

(5) 4CO+SO→42-4CO+2HS+-H+

**TABLE 2:** Products formation from CO by T-Syn enrichment culture, with **(A)** and without **(B)** sulfate.

(A)					

CO concentration (%) (*P*_total_ = 170 kPa)	20	40	60	80	100
Incubation time (days)	11	15	32	49	99
Acetate production (mM)	1.1 ± 0.2	4.4 ± 0.7	8.6 ± 1.5	11.5 ± 1.5	12.5 ± 2.2
H_2_ production (mmol L^–1^_medium_)	2.3 ± 3.6	0.3 ± 0.4	4.9 ± 8.1	0.3 ± 0.2	0.1 ± 0.0
*CO_2_ production (mmol L^–1^_medium_)	7.6 ± 2.1	11.7 ± 1.2	29.3 ± 4.5	22.5 ± 4.2	18.2 ± 3.2
Carbon recovery (%)	77.5 ± 9.8	71.0 ± 4.7	105.0 ± 7.9	85.8 ± 4.7	87.0 ± 1.8
Sulfate utilization (mM)	14.8	7.6	3.8	1.5	1.0

**(B)**					

**CO concentration (%) (*P*_total_ = 170 kPa)**	**20**	**40**	**60**	**80**	**100**

Incubation time (days)	11	15	32	49	99
Acetate production (mM)	0.7 ± 8.9	1.9 ± 1.0	3.4 ± 0.4	5.9 ± 1.8	7.4 ± 2.9
H_2_ production (mmol L^–1^_medium_)	10.6 ± 1.1	21.5 ± 0.6	27.3 ± 0.5	37.9 ± 5.5	0.2 ± 0.1
*CO_2_ production (mmol L^–1^_medium_)	10.6 ± 0.4	18.9 ± 2.0	30.6 ± 18.1	33.7 ± 0.8	11.2 ± 2.7
Carbon recovery (%)	81.5 ± 8.2	72.3 ± 4.4	87.8 ± 43.7	93.2 ± 11.2	79.6 ± 3.3

Sulfate utilization is presented in **(A)**. *Total CO_2_ was estimated by the sum of the gaseous CO_2_ measurement and dissolved CO_2_ calculated using the Henry law. Concentration of gases is expressed as mmol per liter of medium.

The results also showed that sulfate utilization by T-Syn culture was reduced at higher CO concentrations (Table 2A), suggesting an inhibition of the enzymes responsible for sulfate reduction by high concentrations of carbon monoxide. Similar results were obtained when studying sulfate reduction by *D. kuznetsovii* and *D. thermobenzoicus* subsp. *thermosyntrophicus*, which was also partially inhibited at high CO concentrations (Parshina et al., 2005a).

### Carbon Monoxide Conversion by *Desulfofundulus australicus* Strain AB33^T^

**FIGURE 2:**
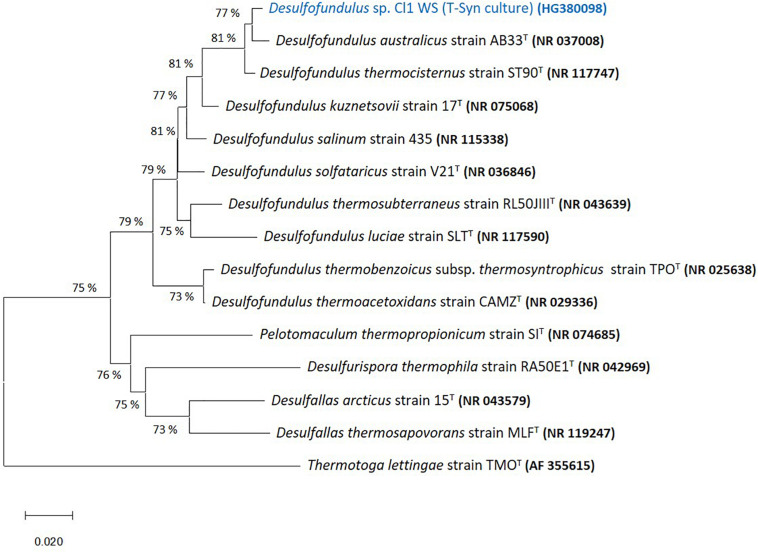
Neighbor joining tree based on 16S rRNA sequences showing the phylogenetic affiliation of the predominant *Desulfofundulus* sp. in T-Syn culture (highlighted in blue) and distance to some of its closest relatives. The evolutionary history was inferred using the Neighbor-joining method (Saitou and Nei, 1987). The optimal tree with the sum of branch length = 0.56105798 is shown. The tree is drawn to scale, with branch lengths in the same units as those of the evolutionary distances used to infer the phylogenetic tree. The evolutionary distances were computed using the p-distance method (Nei and Kumar, 2000) and are in the units of the number of base differences per site. All ambiguous positions were removed for each sequence pair (pairwise deletion option). Evolutionary analyses were conducted in MEGA X (Kumar et al., 2018). GenBank accession numbers of 16S rRNA gene sequences are indicated in parentheses. *Thermotoga lettingae* strain TMO^T^ (AF355615) was used as an outgroup. Bar, 2% estimated difference in nucleotide sequence position.

The 16S rRNA gene sequence of predominant organisms from T-Syn culture (100% CO in the headspace; 170 kPa) is represented in a phylogenetic tree together with its closest relatives of genera belonging to *Peptococcaceae* family, namely, *Desulfofundulus australicus* strain AB33^T^ (98.4% 16S rRNA gene sequence identity), *D. thermocisternus* (98.3%), and *D. kuznetsovii* (95.3%), among others (Figure 2).

**FIGURE 3:**
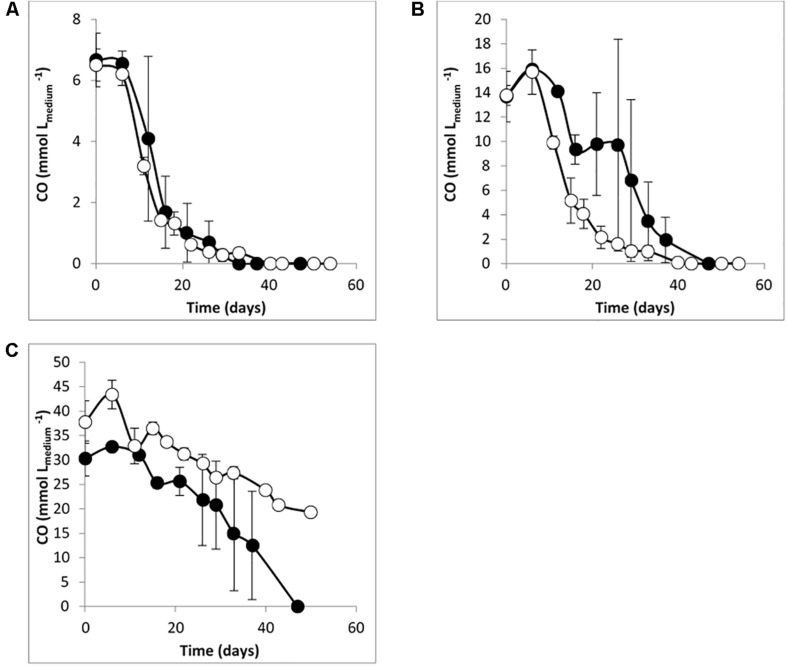
CO degradation by *Desulfofundulus australicus* strain AB33^T^ with **(A)** 10%, **(B)** 20%, and **(C)** 40% of CO, *P*_total_ = 170 kPa. Open symbols: medium without sulfate; filled symbols: medium with sulfate (≈ 20 mM). The bars indicate maximum and minimum values measured (*n* = 3).

*Desulfofundulus australicus* strain AB33^T^ is a thermophilic SRB that can grow autotrophically with H_2_/CO_2_ with sulfate (Love et al., 1993), but growth with CO was never studied. Therefore, the ability of *D. australicus* strain AB33^T^ to grow with CO as sole carbon and energy source was investigated. This strain was able to use up to 40% CO (170 kPa) in the presence of sulfate as electron acceptor (no growth was observed with higher CO concentrations). Contrary to what was observed for culture T-Syn, CO conversion by *D. australicus* strain AB33^T^ in the absence of sulfate was slower than with sulfate, and CO was not completely converted at the end of the incubation (Figure 3). Generally, conversion of CO by culture T-Syn was faster than by *D. australicus* strain AB33^T^, which may be due to previous growth of culture T-Syn with syngas and adaptation to CO. As an example: in the absence of sulfate, T-Syn culture was able to convert 20% CO (170 kPa) in 13 days, while for *D. australicus* strain AB33^T^, 40 days were needed to convert the same amount of CO. *D. australicus* strain AB33^T^ did not produce H_2_ from CO, both in the presence and in the absence of sulfate, while in T-Syn culture H_2_ was produced in both conditions (Table 2).

### Comparison of the MAG of *Desulfofundulus* sp. T-Syn and the Genome of *D. australicus* Strain AB33^T^

**FIGURE 4:**
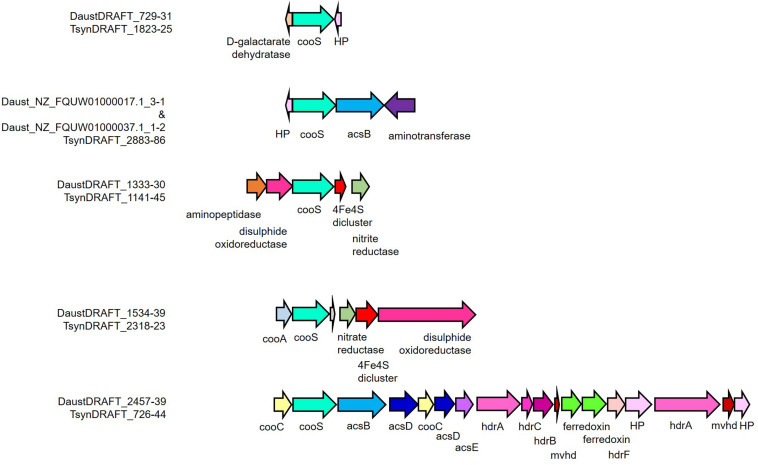
Organization of cooS and neighboring genes in the *D. australicus* and T-Syn draft genomes. acsB, acetyl-CoA synthase catalytic subunit; acsC, corrinoid iron–sulfur protein large subunit; acsD, corrinoid iron-sulfur protein small subunit; acsE, methyl-tetrahydrofolate methyltransferase; amt, aminotransferase; cooA, carbon monoxide dehydrogenase transcriptional regulator; cooC, carbon monoxide dehydrogenase maturation factor; cooS, carbon monoxide dehydrogenase catalytic subunit; HP, hypothetical protein; mvhd, methylviologen-reducing hydrogenase.

Genome analysis of *D. nigrificans* strain CO-1-SRB and the *D. nigrificans*-type strain suggested that the hydrogenogenic carboxydotrophic ability of strain CO-1-SRB is associated with the occurrence of an additional cooS gene that was located next to hydrogenase genes in the genome (Visser et al., 2014). These genes showed high similarity to the carbon monoxide dehydrogenase/energy-converting hydrogenase (CODH/ECH) complex found in *Carboxydothermus hydrogenoformans*, a non-sulfate-reducing thermophile that is able to grow hydrogenogenically with 100% CO (Wu et al., 2005). A cooS gene encodes the catalytic subunit of the anaerobic CODH, and the genes surrounding the cooS gene can indicate to which function the oxidation of CO is coupled. Different functions have been described but also predicted, for example energy conservation, carbon fixation, decreasing oxidative stress, and reduction of ferredoxin and NADP+ (Wu et al., 2005; Techtmann et al., 2012; Diender et al., 2015). A draft genome of *D. australicus* strain AB33^T^ is publicly available at the Integrated Microbial Genomes and Microbiomes server (Joint Genome Institute, Walnut Creek, CA, United States). The metagenome of T-Syn was sequenced, and a single high-quality metagenome-assembled genome (MAG) could be obtained (*Desulfofundulus* sp.) (completeness and contamination – 99.37% and 0.84%, respectively). The MAG of *Desulfofundulus* sp. T-Syn and the genome of *D. australicus* strain AB33^T^ had an average nucleotide identity (ANI) of shared genes above 99%. This ANI value is much higher than the 95% value corresponding to the 70% DNA–DNA hybridization level for species discrimination, indicating that the predominant *Desulfofundulus* in T-Syn culture is a strain of *D. australicus*. To reveal the potential genomic similarities and differences between *Desulfofundulus* sp. T-Syn and *D. australicus* strain AB33^T^, a bidirectional BLAST of the MAG/genome was performed. The genomes of the *Desulfofundulus* sp. T-Syn and *D. australicus* strain AB33^T^ were very similar; from the 3023 detected genes in the T-syn assembly, only 32 genes were not found in *D. australicus*. Their highest similarity was to genes from *D. kuznetsovii*, *Desulfallas geothermicus*, *Desulfofundulus thermosubterraneus*, *Desulfotomaculum hydrothermale*, *Desulfitobacterium hafniense*, *M. thermoacetica*, *Calderihabitans maritimus*, *Tepidanaerobacter acetatoxydans*, *Caloramator australicus*, *Mailhella massiliensis*, *Thermoanaerobacterales* bacterium, and *Xylella fastidiosa*. Moreover, 48 genes were unique in *D. australicus* strain AB33^T^ (Supplementary Table 1). Both T-Syn and *D. australicus* strain AB33^T^ have five cooS genes with similar neighbor genes/synteny (Figure 4). However, the acsB genes from the public assembly (Daust_NZ_FQUW01000070.1_1, Daust_NZ_FQUW01000047.1_1, Daust_NZ_FQUW0100003 7.1_1, Daust_NZ_FQUW01000031.1_1, and Daust_NZ_FQU W01000017.1_1) were all on the edge of a scaffold. Therefore, they are all smaller than the T-Syn homologs. The four parts are derived from two genes split by the assembly. Which parts belonged to each acsB genes could be inferred after analyzing the continuity of the synteny. The acsB that belongs to the operon structure of the acetyl-CoA pathway (in T_syn: T_synDRAFT_0728) is divided in NZ_FQUW01000047.1_1 (13%; percentage of gene length) and NZ_FQUW01000031.1_1 (27%), while the other gene (in T-Syn: T_synDRAFT_2885) is divided in Daust_NZ_FQUW01000017.1_1 (16%) and Daust_NZ_FQUW01000037.1_1 (4%). The remaining partial acsB Daust_NZ_FQUW01000070.1_1 covers the gene for 80% and is the only (partial) gene of the scaffold. In the *D. australicus* strain AB33^T^ assembly, the full acetyl-CoA pathway is in one scaffold (DaustDRAFT_2457-2439), including the complete acsB gene, which indicates that there is no difference between the acetyl-CoA pathway of *D. australicus* strain AB33^T^ and T-Syn. However, the cooS with acsB (for T-Syn: 2884-2885) are missing in the *D. australicus* strain AB33^T^ assembly. The physiological differences (higher CO tolerance and hydrogen production) between the *D. australicus* strain AB33^T^ and the *Desulfofundulus* sp. T-Syn cannot be directly linked to the genomic comparison. The fact that *Desulfofundulus australicus* T-syn has been subjected to an adaptation period during the enrichment process in syngas (CO-rich gas) could play a role in the higher performance during CO conversion. Adaptation to CO has been observed in other carboxydotrophs, as for example in *Thermoanaerobacter kivui* (Weghoff and Müller, 2016). *T. kivui* could be adapted to grow on CO (even within a 100% CO atmosphere) after an adaptation process by increasing the CO concentration in small increments (10%) (Weghoff and Müller, 2016).

## Conclusion

This work contributes for the knowledge on thermophilic carboxydotrophic organisms, specifically, thermophilic SRB that are able to grow and use C1 compounds as carbon and energy source. Even though the growth of most SRB is generally described as being inhibited by CO, there are several thermophilic sulfate reducers, such as the *D. australicus* species investigated in this work that can grow well at high CO concentrations. From a syngas-degrading enrichment culture, it was possible to obtain a highly enriched culture, mainly composed of *Desulfofundulus*-related organisms that are able to use CO at high concentrations. The products formed from CO conversion were H_2_ and acetate. When supplied to the cultures, sulfate was reduced to sulfide, and a lesser amount of H_2_ was formed in this condition. This work also reports the ability of *D. australicus* strain AB33^T^ to use CO up to 40% (170 kPa) as sole carbon and energy source. The high CO tolerance and/or the ability of some SRB to use CO, e.g., thermophilic *Desulfofundulus* species, opens perspectives for their biotechnological application, such as using CO-rich syngas as a cheap alternative electron donor for sulfate reduction in bioremediation processes.

## Data Availability Statement

Nucleotide sequences (16S rRNA gene sequences) described within this study have been submitted to the European Nucleotide Archive (ENA) under accession numbers HG380098 and HG380099. FASTQ files and raw sequencing data were submitted to ENA under BioProject accession number PRJEB34645 (https://www.ebi.ac.uk/ena/browser/view/PRJEB34645).

## Author Contributions

JA and DS: conceptualization and investigation. JA, MV, and DS: methodology. JA, MV, AA, and BN: formal analysis. MV and BN: data curation. JA: writing – original draft preparation. JA, MV, AA, BN, CP, MA, AS, and DS: writing – review and editing. DS, CP, AS, and MA: supervision. All authors have read and agreed with the published version of the manuscript.

## Conflict of Interest

The authors declare that the research was conducted in the absence of any commercial or financial relationships that could be construed as a potential conflict of interest.

## Footnotes

1 http://www.ncbi.nlm.nih.gov/blast/
