# Supplementary material for: Effect of Sulfate on Carbon Monoxide Conversion by a Thermophilic Syngas-Fermenting Culture Dominated by a Desulfofundulus Species
Source: Front Microbiol. 2020 Nov 16;11:588468. doi: 10.3389/fmicb.2020.588468 (PMC7701048; doi:10.3389/fmicb.2020.588468)
Supplement: Supplementary file 1 [file Data_Sheet_1.docx]

**Effect of sulfate on carbon monoxide conversion by a thermophilic syngas-fermenting culture dominated by a *Desulfofundulus* species**

**Joana I. Alves^1^, Michael Visser^2+^, Ana L. Arantes^1^, Bart Nijsse^3^, Caroline M. Plugge^2^, M. Madalena Alves^1^, Alfons J. M. Stams^1,2^ and Diana Z. Sousa^1,2^***

^1^Centre of Biological Engineering, University of Minho, Braga, Portugal

^2^Laboratory of Microbiology, Wageningen University & Research, Wageningen, The Netherlands

^3^Laboratory of Systems and Synthetic Biology, Wageningen University & Research, Wageningen, The Netherlands

^+^ Current adress: Dutch National Plant Protection Organization, National Reference Centre, Wageningen, The Netherlands

*** Correspondence:**Diana Z. Sousa

diana.sousa@wur.nl

Supplementary Material

**Supplementary Table 1.**

Supplementary Table 1 – Main genomic differences between *D. australicus* strain AB33^T^ and T-Syn

| **Genes that did not have Daust as best hit** | **Putative Function** | **Species** | **Identities (%)** |
| --- | --- | --- | --- |
| Tsyn_1472 | hypothetical protein XD63_1795 | *Thermoanaerobacterales* bacterium 50_218 | 99% |
| Tsyn_1535 | hypothetical protein XD63_1795 | *Thermoanaerobacterales* bacterium 50_218 | 99% |
| Tsyn_1642 | electron transfer flavoprotein beta subunit/FixA family protein | *Desulfofundulus thermocisternus* | 99% |
| Tsyn_1816 | hypothetical protein XD63_1795 | *Thermoanaerobacterales* bacterium 50_218 | 99% |
| Tsyn_2000 | electron transfer flavoprotein beta subunit/FixA family protein | *Desulfofundulus thermocisternus* | 99% |
| Tsyn_2121 | hypothetical protein XD63_1795 | *Thermoanaerobacterales* bacterium 50_218 | 99% |
| **Genes not present in Daust** | **Putative Function** | **Species** | **Identities (%)** |
| Tsyn_0097 | WP_011391832.1 ATP-binding protein | *Moorella thermoacetica* | 93% |
| Tsyn_0098 | WP_092488071.1 IS21 family transposase | *Desulfofundulus thermocisternus* | 92% |
| Tsyn_0114 | AEG14419.1 transposase, IS605 OrfB family | *Desulfofundulus kuznetsovii* | 94% |
| Tsyn_0347 | AEG15374.1 RNA-directed DNA polymerase (Reverse transcriptase) | *Desulfofundulus kuznetsovii* DSM 6115 | 96% |
| Tsyn_0409 | GAW92296.1 RNA-directed DNA polymerase, partial | *Calderihabitans maritimus* | 94% |
| Tsyn_0558 | AEE92484.1 transposase IS3/IS911 family protein | *Tepidanaerobacter acetatoxydans* | 69% |
| Tsyn_0849 | AEE92484.1 transposase IS3/IS911 family protein | *Tepidanaerobacter acetatoxydans* | 69% |
| Tsyn_1075 | AEE92484.1 transposase IS3/IS911 family protein | *Tepidanaerobacter acetatoxydans* | 69% |
| Tsyn_1434 | AEG14415.1 transposase IS116/IS110/IS902 family protein | *Desulfofundulus kuznetsovii* | 100% |
| Tsyn_1435 | CCJ32921.1 hypothetical protein CAAU_0837 | *Caloramator australicus* RC3 | 58% |
| Tsyn_1474 | AEG14415.1 transposase IS116/IS110/IS902 family protein | *Desulfofundulus kuznetsovii* | 100% |
| Tsyn_1542 | GAW29837.1 hypothetical protein ULO1_24070 | *Carboxydocella* sp. ULO1 | 52% |
| Tsyn_1543 | BAE84557.1 hypothetical protein DSY2768 | *Desulfitobacterium hafniense* Y51 | 44% |
| Tsyn_1544 | WP_083717025.1 response regulator | *Mailhella massiliensis* | 59% |
| Tsyn_1610 | AEG16981.1 transposase IS66 | *Desulfofundulus kuznetsovii* DSM 6115 | 98% |
| Tsyn_1611 | KUK30948.1 Transposase IS66 | *Thermoanaerobacterales* bacterium 50_218 | 99% |
| Tsyn_1615 | OIQ59998.1 putative transposase, YhgA-like | *Moorella thermoacetica* | 55% |
| Tsyn_1616 | WP_011391832.1 ATP-binding protein | *Moorella thermoacetica* | 95% |
| Tsyn_1763 | AEE92484.1 transposase IS3/IS911 family protein | *Tepidanaerobacter acetatoxydans* | 69% |
| Tsyn_1819 | AEG14415.1 transposase IS116/IS110/IS902 family protein | *Desulfofundulus kuznetsovii* | 100% |
| Tsyn_1836 | CCO07876.1 transposase (fragment) | *Desulfotomaculum hydrothermale* Lam5 | 75% |
| Tsyn_1837 | WP_092488071.1 IS21 family transposase | *Desulfallas geothermicus* | 92% |
| Tsyn_1838 | WP_011391832.1 ATP-binding protein | *Moorella thermoacetica* | 93% |
| Tsyn_1846 | AEG15255.1 hypothetical protein Desku_1677 | *Desulfofundulus kuznetsovii* | 98% |
| Tsyn_1883 | AEE92484.1 transposase IS3/IS911 family protein | *Tepidanaerobacter acetatoxydans* | 69% |
| Tsyn_2173 | SHJ40515.1 hypothetical protein SAMN02745219_02485 | *Desulfofundulus thermosubterraneus* | 97% |
| Tsyn_2351 | AEG14419.1 transposase, IS605 OrfB family | *Desulfofundulus kuznetsovii* | 94% |
| Tsyn_2507 | SHI72759.1 Methyl-viologen-reducing hydrogenase, delta subunit | *Desulfofundulus kuznetsovii* | 90% |
| Tsyn_2526 | AIC12375.1 YccS/YhfK family integral membrane protein | *Xylella fastidiosa* | 53% |
| Tsyn_2703 | WP_083717025.1 response regulator | *Mailhella massiliensis* | 59% |
| Tsyn_2776 | WP_011391832.1 ATP-binding protein | *Moorella thermoacetica* | 94% |
| Tsyn_2777 | WP_092488071.1 IS21 family transposase | *Desulfallas geothermicus* | 91% |
